# High-Level HOOK3 Expression Is an Independent Predictor of Poor Prognosis Associated with Genomic Instability in Prostate Cancer

**DOI:** 10.1371/journal.pone.0134614

**Published:** 2015-07-31

**Authors:** Nathaniel Melling, Levon Harutyunyan, Claudia Hube-Magg, Martina Kluth, Ronald Simon, Patrick Lebok, Sarah Minner, Maria Christina Tsourlakis, Christina Koop, Markus Graefen, Meike Adam, Alexander Haese, Corinna Wittmer, Stefan Steurer, Jakob Izbicki, Guido Sauter, Waldemar Wilczak, Thorsten Schlomm, Till Krech

**Affiliations:** 1 Institute of Pathology, University Medical Center Hamburg-Eppendorf, Germany; 2 General, Visceral and Thoracic Surgery Department and Clinic, University Medical Center Hamburg-Eppendorf, Germany; 3 Martini-Clinic, Prostate Cancer Center, University Medical Center Hamburg-Eppendorf, Germany; 4 Department of Urology, Section for translational Prostate Cancer Research, University Medical Center Hamburg-Eppendorf, Germany; Innsbruck Medical University, AUSTRIA

## Abstract

Hook microtubule-tethering protein 3 (HOOK3) is an adaptor protein for microtubule-dependent intracellular vesicle and protein trafficking. In order to assess the role of HOOK3 in prostate cancer we analyzed HOOK3 expression by immunohistochemistry on a TMA containing more than 12,400 prostate cancers. Results were compared to tumor phenotype and PSA recurrence as well as aberrations possibly defining relevant molecular subtypes such as ERG status and deletions of 3p13, 5q21, 6q15 and PTEN. HOOK3 immunostaining was negative in normal luminal cells of prostate epithelium, whereas 53.3% of 10,572 interpretable cancers showed HOOK3 expression, which was considered low in 36.4% and high in 16.9% of cases. High-level HOOK3 expression was linked to advanced tumor stage, high Gleason score, high proliferation index, positive lymph node stage, and PSA recurrence (p<0.0001 each). The prognostic role of HOOK3 expression was independent of established clinico-pathological parameters both in preoperative and postoperative settings. Comparisons with molecular features were performed to draw conclusions on the potential function of HOOK3 in the prostate. A strong association with all examined deletions is consistent with a role of HOOK3 for maintaining genomic integrity by contributing to proper centrosome assembly. Finding HOOK3 expression in 74% of ERG positive but in only 38% of ERG negative cancers (p<0.0001) further suggests functional interactions between these genes. In conclusion, the results of our study identify HOOK3 as a strong candidate prognostic marker with a possible role in maintaining genomic integrity in prostate cancer, which may have potential for inclusion into clinical routine assays.

## Introduction

Prostate cancer is the most prevalent cancer in men in Western societies [1]. While most tumors have a rather indolent clinical course, prostate cancer still represents the third most common cause of cancer related death in men. Established prognostic parameters are Gleason grade, tumor extent on biopsies, preoperative prostate-specific antigen (PSA), and clinical stage. Although statistically powerful, they are not sufficient for optimal individual treatment decisions. It is hoped that a better understanding of disease biology will eventually lead to the identification of clinically applicable molecular markers that enable a more reliable prediction of prostate cancer aggressiveness in individual patients.

The family of human hook microtubule-tethering proteins (HOOKs) comprises three homologues, HOOK1, HOOK2, and HOOK3, which are abundantly expressed in human cells. HOOKs function as adaptor proteins involved in trafficking of membrane vesicles and protein complexes along microtubules between the Golgi apparatus, centrosomes [2–5], endosomes [6] and lysosomes [7]. A cancer relevant role has been suggested specifically for HOOK3 from several studies finding recurrent alterations of the gene. For example, one study reported a HOOK3:RET fusion in a case of papillary thyroid cancer, which proved to be oncogenic in a mouse xenograft cancer model [8]. Another study found protein-altering mutations with unknown significance in 2 of 48 small intestine neuroendocrine tumors [9]. In addition, HOOK3 gene is located at 8p11, a common breakpoint in many human tumor types, including prostate cancer [10,11]. Accordingly, inactivating breakage resulting in reduced expression of HOOK3 was found in a considerable fraction (9.0%) of tumors [11] in a study on 77 prostate cancers.

These findings prompted us to study the patterns of HOOK3 expression in prostate cancer in more detail. We took advantage of our preexisting tissue microarray (TMA) containing >12,000 prostate cancer specimens connected to a database with clinical follow up and extensive molecular data. Our findings demonstrate that high levels of HOOK3 protein expression are strongly linked to adverse tumor phenotype and early PSA recurrence and can independently predict poor outcome in prostate cancer.

## Materials and Methods

### Patients

Radical prostatectomy specimens were available from 12,427 patients, undergoing surgery between 1992 and 2012 at the Department of Urology and the Martini Clinics at the University Medical Center Hamburg-Eppendorf. Follow-up data were available for a total of 12,344 patients with a median follow-up of 36 months (range: 1 to 241 months; Table 1). Prostate specific antigen (PSA) values were measured following surgery and PSA recurrence was defined as the time point when postoperative PSA was at least 0.2ng/ml and increasing at subsequent measurements. All prostate specimens were analyzed according to a standard procedure, including a complete embedding of the entire prostate for histological analysis [12]. The TMA manufacturing process was described earlier in detail [13]. In short, one 0.6mm core was taken from a representative tissue block from each patient. The tissues were distributed among 27 TMA blocks, each containing 144 to 522 tumor samples. For internal controls, each TMA block also contained various control tissues, including normal prostate tissue. The molecular database attached to this TMA contained results on ERG expression in 10,678 [14], ERG break apart FISH analysis in 7,099 (expanded from [15]), deletion status of 5q21 (CHD1) in 7,932 (expanded from [16]), 6q15 (MAP3K7) in 6,069 (expanded from [17]), PTEN (10q23) in 6,704 (expanded from [18]), 3p13 (FOXP1) in 7,081 (expanded from [19]) cancers, and Ki67 labeling index (Ki67LI) data in 4,426 cancers (expanded from [20]).

**Table 1:** Pathological and clinical data of the arrayed prostate cancers.

	No. of patients (%)	
Parameter	Study cohort on TMA(n = 12,427)	Biochemical relapse among categories
**Follow-up (month)**		
n	11,665 (94)	2,769 (24)
Mean	48.9	-
Median	36.4	-
**Age (y)**		
≤50	334 (3)	81 (24)
51–59	3,061 (25)	705 (23)
60–69	7,188 (58)	1,610 (22)
≥70	1,761 (14)	370 (21)
**Pretreatment PSA (ng/ml)**		
<4	1,585 (13)	242 (15)
4–10	7,480 (61)	1,355 (18)
>10–20	2,412 (20)	737 (31)
>20	812 (7)	397 (49)
**pT stage**		
pT2	8,187 (66)	1,095 (13)
pT3a	2,660 (22)	817 (31)
pT3b	1,465 (12)	796 (54)
pT4	63 (1)	51 (81)
**Gleason grade**		
≤3+3	2,983 (24)	368 (12)
3+4	6,945 (56)	1,289 (19)
4+3	1,848 (15)	788 (43)
≥4+4	584 (5)	311 (53)
**pN stage**		
pN0	6,970 (91)	1,636 (24)
pN+	693 (9)	393 (57)
**Surgical margin**		
Negative	9,990 (82)	1,848 (19)
Positive	2,211 (18)	853 (39)

Percentage in the column “Study cohort on TMA” refers to the fraction of samples across each category. Percentage in column “Biochemical relapse among categories” refers to the fraction of samples with biochemical relapse within each parameter in the different categories. Abbreviation: TMA tissue micro array

### Ethics statement

The usage of archived diagnostic left-over tissues for manufacturing of tissue microarrays and their analysis for research purposes as well as patient data analysis has been approved by local laws (HmbKHG, §12,1) and by the local ethics committee (Ethics commission Ärztekammer Hamburg, WF-049/09 and PV3652). According to local laws, informed consent was not required for this study. Patient records/information was anonymized and de-identified prior to analysis. All work has been carried out in compliance with the Helsinki Declaration.

### Immunohistochemistry

Freshly cut TMA sections were immunostained on one day and in one experiment. Slides were deparaffinized and exposed to heat-induced antigen retrieval for 5 minutes in an autoclave at 121°C in pH 7.8 Tris-EDTA-Citrate buffer. Affinity purified primary antibody HPA024756 raised against the protein HOOK homolog 3 recombinant protein epitope signature tag (KEEIAQRCHELDMQVAALQEEKSSLLAENQVLMERLNQSDSIEDPNSPAGRRHLQLQTQLEQLQEETFRLEA) (rabbit polyclonal antibody, Sigma, St. Louis, MO, USA; dilution 1:150) was applied at 37°C for 60 minutes. Specificity of the antibody was validated by the manufacturer, as it showed a single band at the predicted size (83.1 kD) in a western blot. Bound antibody was then visualized using the EnVision Kit (Dako, Glostrup, Denmark) according to the manufacturer´s directions [15]. The antibody usually stained the tumor cell cytoplasm in all cells (100%) of a given tissue spot. Staining intensity of all cases was thus semiquantitatively assessed in three categories: negative, low (weak to moderate) and high (strong staining intensity).

### Statistics

Statistical calculations were performed with JMP 10.0.2 software (SAS Institute Inc., NC, USA). Contingency tables and the chi²-test were performed to search for associations between molecular parameters and tumor phenotype. Survival curves were calculated according to Kaplan-Meier. The Log-Rank test was applied to detect significant differences between groups. Analysis of variance (ANOVA) test was applied to search for associations between cell proliferation and HOOK3 staining. Cox proportional hazards regression analysis was performed to test the statistical independence and significance between pathological, molecular and clinical variables. Separate analyses were performed using different sets of parameters available either before or after prostatectomy.

## Results

### Technical aspects

A total of 10,572 (85.0%) of tumor samples were interpretable in our TMA analysis. Reasons for non-informative cases (1,855 spots; 15.0%) included lack of tissue spots in the TMA section or absence of unequivocal cancer tissue in the TMA spot.

### HOOK3 expression in normal and cancerous prostatic cells

**Fig 1:**
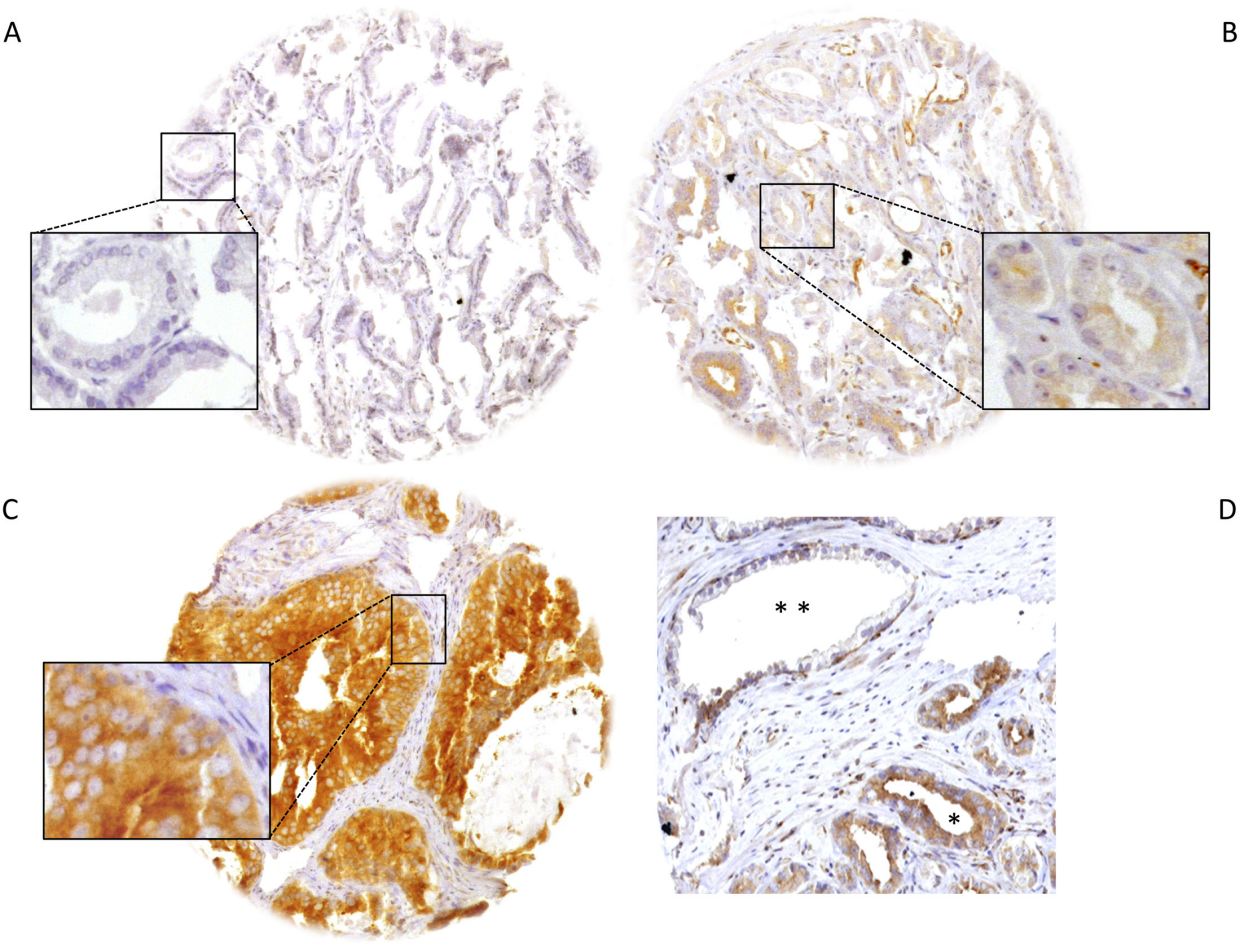
Representative pictures of HOOK3 immunostaining in prostate cancer. (a) negative, (b) low intensity, (c) high intensity staining at 10x, insets at 300x magnification and (d) negative normal luminal cells and positive basal cells (*) together with high-level positive cancerous tissue (**) in the same core at 150x magnification.

Representative images of negative and positive HOOK3 immunostainings are given in Fig 1. HOOK3 immunostaining was localized in the cytoplasm of cells (Fig 1c insert). Normal tissues, derived from prostate cancer patients, showed no staining of stromal and luminal cells, while basal cells stained positive (** in Fig 1d). Positive HOOK3 immunostaining was seen in 5,636 of our 10,572 (53.3%) interpretable prostate cancers and was considered low in 36.4% (Fig 1b) and high in 16.9% of cancers (Fig 1c).

### Association with TMPRSS2:ERG fusion status and ERG protein expression

**Fig 2:**
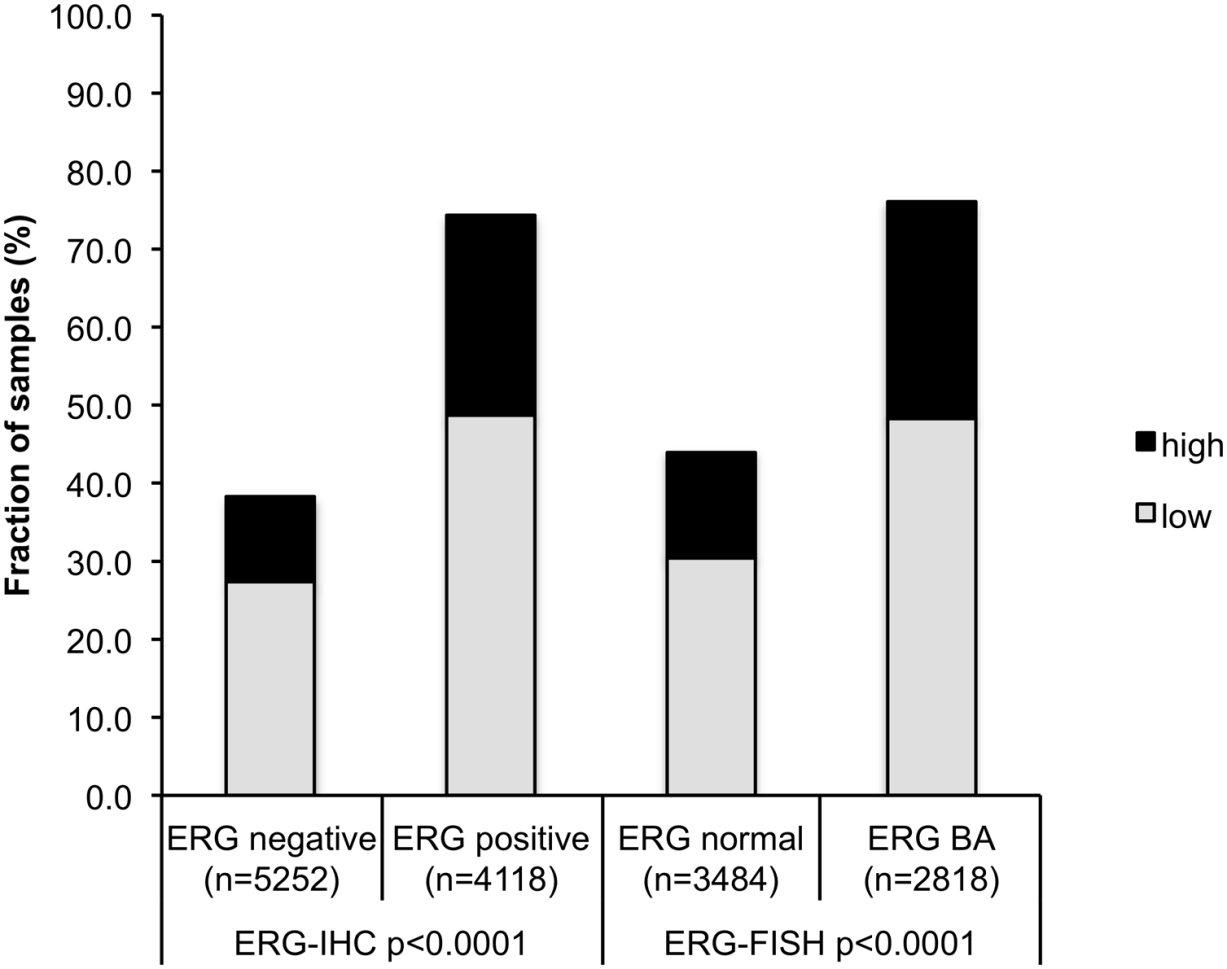
Relationship of HOOK3 expression with ERG status. IHC = immunohistochemistry; FISH = fluorescence in-situ hybridization.

To evaluate whether HOOK3 expression is associated with the TMPRSS2:ERG fusion in prostate cancers, we used data from previous studies (expanded from [14,15]. Data on TMPRSS2:ERG fusion status obtained by FISH were available from 6,302 and by immunohistochemistry from 9,370 tumors with evaluable HOOK3 immunostaining. Data on both ERG FISH and IHC were available from 6076 cancers, and an identical result (ERG IHC positive and break by FISH or ERG IHC negative and missing break by FISH) was found in 5,811 of 6,076 (95,6%) cancers. Positive HOOK3 immunostaining was linked to TMPRSS2:ERG rearrangement and ERG positivity in prostate cancers. HOOK3 immunostaining was seen in 74.3% and 76.1% of cancers with TMPRSS2:ERG fusion detected by IHC and FISH, but found in only 38.2% of cancers without ERG staining and 44.1% of cancers without ERG rearrangements detected by FISH (p<0.0001 each, Fig 2).

### Association with tumor phenotype

When all the carcinomas were jointly analyzed, high-level HOOK3 expression was significantly linked to advanced pathological tumor stage, high Gleason grade, lymph node metastases (p<0.0001 each) and surgical margin positivity (p = 0.0003). No correlation was found with high preoperative PSA-levels (p = 0.7594; Table 2). Subgroup analysis for ERG-negative and positive cancers revealed similar results (S1 and S2 Tables).

**Table 2:** Clinico-pathological association of HOOK3 immunostaining in prostate cancer.

		HOOK3	staining	(%)	P
Parameter	N	Negative	Low	High	value
**All cancers**	10,572	47	36	17	
**Tumor stage**					<0.0001
pT2	6,853	52	35	14	
pT3a	2,341	40	40	21	
pT3b-pT4	1,337	33	40	28	
**Gleason grade**					<0.0001
≤3+3	2,383	64	29	7.3	
3+4	6,004	46	38	17	
4+3	1,624	31	42	27	
≥4+4	508	31	37	33	
**Lymph node metastasis**					<0.001
N0	5,950	42	38	19	
N+	622	30	39	31	
**Preoperative PSA level (ng/ml)**					0.75
<4	1,289	45	38	17	
4–10	6,349	46	37	17	
10–20	2,090	48	36	17	
>20	729	48	35	18	
**Surgical margin**					0.0003
negative	8,409	47	37	16	
positive	1,971	45	36	20	

### Association with other key genomic deletions

**Fig 3:**
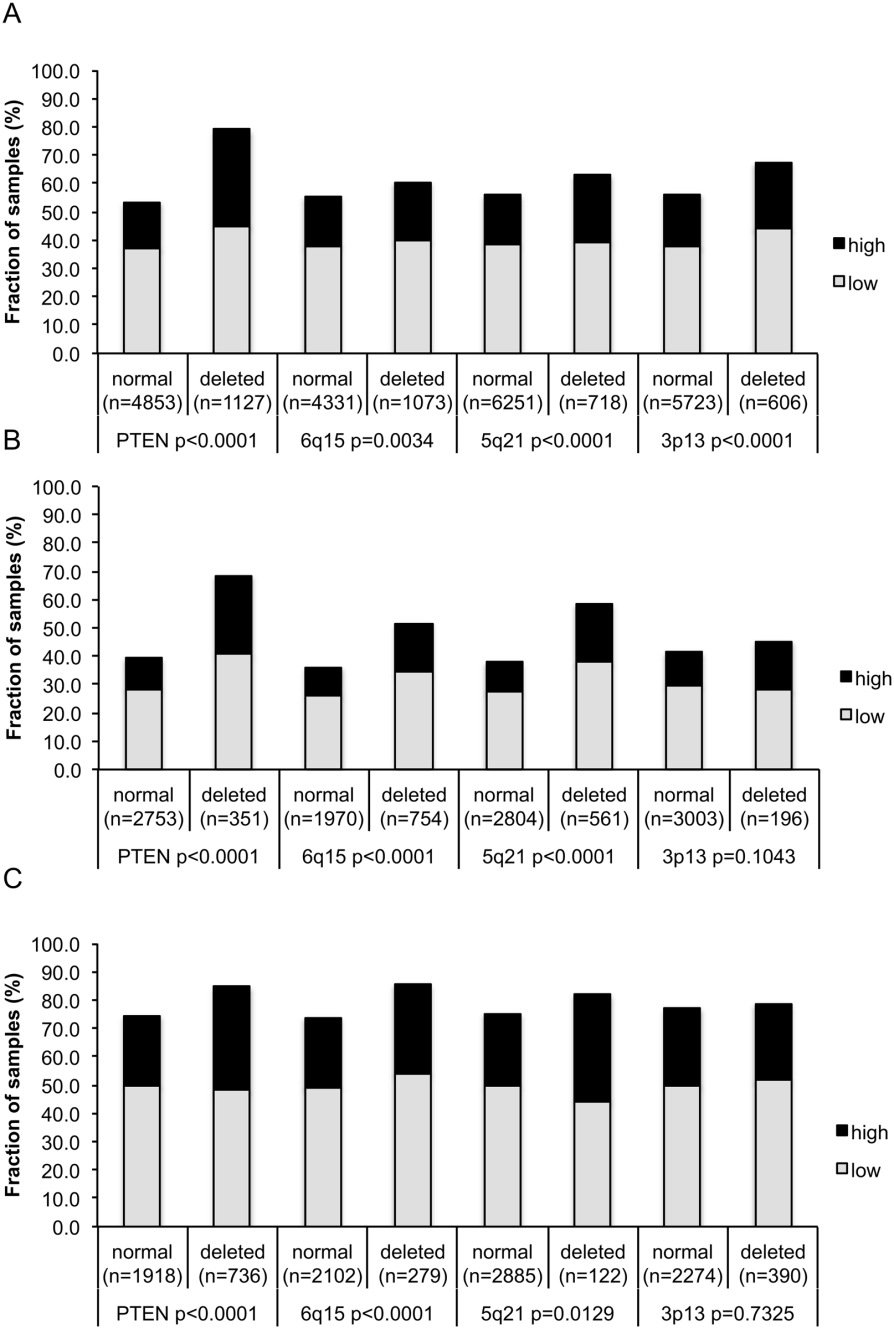
HOOK3 expression versus *PTEN*, 3p13, 6q15 and 5q21 deletions probed by FISH analysis. (a) all cancers, (b) in ERG-negative, c) ERG-positive subset.

Earlier studies have provided evidence for distinct molecular subgroups of prostate cancers defined by TMPRSS2:ERG fusions and several genomic deletions. Others and us have previously described a strong link of PTEN and 3p13 deletions to ERG positivity and of 5q21 and 6q15 deletions to ERG negativity [16–19]. So as to examine, whether HOOK3 expression might be particularly associated with one of these genomic deletions, HOOK3 data were compared to preexisting findings on PTEN (10q23), 3p13 (FOXP1), 6q15 (MAP3K7) and 5q21 (CHD1) deletions. In the analysis of all tumors, HOOK3 expression was significantly linked to all the deletions mentioned above (PTEN, 5q21 and 3p13 (p<0.0001 each), 6q15 (p = 0.003); Fig 3a). These associations varied when subgroup analysis was performed for ERG negative (Fig 3b) and ERG positive cancers (Fig 3c). Here HOOK3 staining was strongly correlated with deletions in PTEN (p<0.0001 for both ERG negative and positive cancers), 6q15 (both p<0.0001) and 5q21 (p<0.0001, p = 0.01 respectively) but not with 3p13 deletions (p = 0.10 and p = 0.73 respectively).

### Association with tumor cell proliferation (Ki67 labeling index)

Strong HOOK3 staining was significantly linked to accelerated cell proliferation as measured by Ki67LI in all cancers as well as in subsets of cancers with identical Gleason score (≤3+3, 3+4, 4+3, and ≥4+4, p<0.0001 each, Table 3).

**Table 3:** Association of HOOK3 immunostaining and Ki67 labeling index.

			Ki67 labeling	index	P
Subset	HOOK3	N	Mean	SD	value
total	negative	2,981	2.1	0.0	
	low	2,327	3.2	0.1	<0.0001
	high	975	3.6	0.1	
ERG-negative	negative	2,168	2.0	0.1	
	low	917	3.4	0.1	<0.0001
	high	317	4.2	0.1	
ERG-positive	negative	769	2.5	0.1	
	low	1,374	3.0	0.1	<0.0001
	high	651	3.3	0.1	
Prostatektomie Gleason ≤3+4	negative	2,567	2.0	0.0	
	low	1,827	3.0	0.1	<0.0001
	high	687	3.2	0.1	
Prostatektomie Gleason ≥4+3	negative	397	2.8	0.2	
	low	487	3.9	0.2	<0.0001
	high	283	4.7	0.2	
PTEN normal	negative	1,553	2.5	0.1	
	low	1,239	3.3	0.1	<0.0001
	high	481	3.8	0.1	
PTEN deletion	negative	157	2.8	0.2	
	low	325	3.8	0.2	0.0002
	high	215	4.1	0.2	

### Association with PSA recurrence

**Fig 4:**
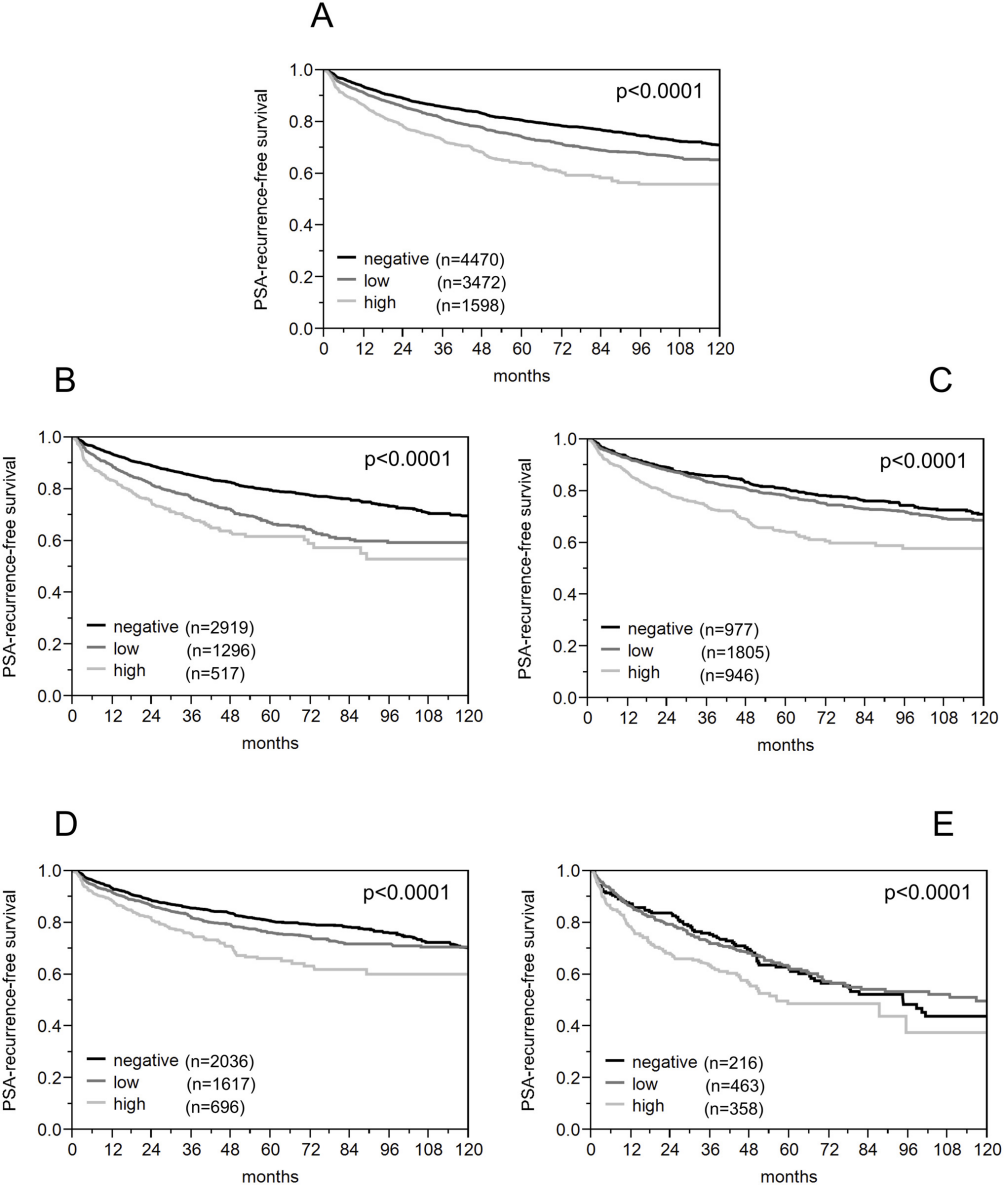
Association of HOOK3 expression with biochemical recurrence. (a) all cancers (n = 9,540), (b) ERG-negative (ERG^-^) cancers (n = 4,732), (c) ERG-positive (ERG^+^) cancers (n = 3,678), (d) *PTEN* non-deleted (PTEN^norm^) cancers (n = 4,349), (e) *PTEN* deleted cancers (n = 1037).

Follow-up data were available for 9,916 patients with interpretable HOOK3 immunostaining on the TMA. A highly significant association between high-level HOOK3 expression and early PSA recurrence was found when all tumors were analyzed and also in the subgroup analyses for ERG negative and positive cancers (p<0.0001 each; Fig 4a–4e).

### Multivariate analysis

Four different types of multivariate analyses were performed evaluating the clinical relevance of HOOK3 expression in different scenarios (Table 4). Scenario 1 evaluated all postoperatively available parameters including pathological tumor stage, pathological lymph node status (pN), surgical margin status, preoperative PSA value and pathological Gleason grade obtained after the morphological evaluation of the entire resected prostate. In scenario 2, all postoperatively available parameters with exception of nodal status were included. The rational for this approach was that the indication and extent of lymph node dissection is not standardized in the surgical therapy of prostate cancer and that excluding pN in multivariate analysis can markedly increase case numbers. Two additional scenarios had the purpose to model the preoperative situation as much as possible. Scenario 3 included HOOK3 expression, preoperative PSA, clinical tumor stage (cT stage) and Gleason grade obtained on the prostatectomy specimen. Since postoperative determination of a tumors Gleason grade is “better” than the preoperatively determined Gleason grade (subjected to sampling errors and consequently under-grading in more than one third of cases [21]), another multivariate analysis was added. In scenario 4, the preoperative Gleason grade obtained on the original biopsy was combined with preoperative PSA, cT stage and HOOK3 expression. HOOK3 proved to be an independent prognostic parameter in all scenarios when all tumors and also subgroups of ERG negative and ERG positive tumors were analyzed (Table 4). HOOK3 proved to be an independent prognosticator irrespective of the tested scenario or subgroup (all cancers: p = 0.0003 in scenario 1, p<0.0001 in scenario 2–4; ERG negative cancers: p = 0.0002 in scenario 1, p<0.0001 in scenario 2–4; ERG positive cancers: p = 0.0381 in scenario 1, p = 0.0433 in scenario 2, p = 0.0006 in scenario 3, p<0.0001 in scenario 4).

**Table 4:** Multivariate analysis of HOOK3 expression in prostate cancer, the ERG-negative and positive subset by immunohistochemistry.

			P value							
Tumor	Scen-		preoperative			Gleason grade	Gleason			HOOK3
subset	ario	N	PSA-level	pT Stage	cT Stage	prostatectomy	Grade biopsy	pN Stage	R Stage	expression
**all cancers**	1	5,056	<0.0001	<0.0001	-	<0.0001	-	<0.0001	0.0005	0.0003
	2	8,085	<0.0001	<0.0001	-	<0.0001	-	-	<0.0001	<0.0001
	3	7,986	<0.0001	-	<0.0001	<0.0001	-	-	-	<0.0001
	4	7,884	<0.0001	-	<0.0001	-	<0.0001	-	-	<0.0001
**ERG-negative subset**	1	2,574	<0.0001	<0.0001	-	<0.0001	-	0.0008	0.13	0.0002
	2	4,019	<0.0001	<0.0001	-	<0.0001	-	-	0.0004	<0.0001
	3	3,987	<0.0001	-	<0.0001	<0.0001	-	-	-	<0.0001
	4	3,933	<0.0001	-	<0.0001	-	<0.0001	-	-	<0.0001
**ERG-positive subset**	1	2,037	0.0002	<0.0001	-	<0.0001	-	0.0100	0.002	0.038
	2	3,199	<0.0001	<0.0001	-	<0.0001	-	-	<0.0001	0.04
	3	3,138	<0.0001	-	<0.0001	<0.0001	-	-	-	0.0006
	4	3,100	<0.0001	-	<0.0001	-	<0.0001	-	-	<0.0001

## Discussion

The results of this study demonstrate that high-level HOOK3 expression is an independent predictor of early PSA recurrence in prostate cancer.

Our immunohistochemical analysis revealed cytoplasmic HOOK3 staining in 85.0% of 10,572 analyzable prostate cancers. Normal prostate epithelium typically showed negative or weak immunostaining in luminal cells, while basal and stromal cells did not stain for HOOK3. That increasing levels of HOOK3 paralleled cancer aggressiveness is consistent with a relevant role of HOOK3 up regulation for prostate cancer development and progression. Data from The Human Protein Atlas (www.proteinatlas.org) seem to suggest that HOOK3 can also be up regulated in other cancer types, including colorectal cancer, endometrial cancer, glioma, lung cancer, lymphoma, and thyroid cancer [22].

The strong association of high-level HOOK3 expression with adverse tumor features, including advanced stage, high Gleason grade, nodal metastasis and PSA recurrence argues for a practical relevance of HOOK3 measurement for prognosis assessment. That strong HOOK3 expression pertained prognostic relevance even in the subset of cancers harboring PTEN deletions—one of the strongest known prognostic markers in prostate cancer [18,23,24]–further emphasizes a clinically relevant role of HOOK3 testing. This is all the more true as the prognostic impact of HOOK3 was also independent of clinical and pathological parameters. Our extensive multivariate modeling further suggests that HOOK3 might be a clinically useful prognostic marker in both pre- and postoperative scenarios. Considering that a clinical biomarker must be analyzed on biopsy material and before treatment decisions are taken, it is of note, that our approach of analyzing molecular features on one minute TMA tissue specimen measuring 0.6 mm in diameter closely models the molecular analyses of core needle biopsies where comparable amounts of tissues are evaluated. As our TMA samples were not exactly taken from the “worst” area of each tumor but randomly from within a representative cancer area, our TMA spot might be as representative as possible of the “worst” area of a clinical cancer identified in a set of cancer biopsies.

It is unknown how HOOK3 may contribute to cancer development and progression. We did not perform own functional experiments, but the large number of prostate cancers included in our project together with extensive molecular information on our tumors enabled us to draw some conclusions on putative cancer-relevant roles of HOOK3 “in silico”. This approach of “functional molecular epidemiology” first demonstrated, that HOOK3 expression is strongly linked to classical parameters of genomic instability, such as prevalence of chromosomal deletions, and to elevated cell proliferation. Deletions of certain small and large chromosomal regions are a hallmark of prostate cancer. Data from next generation sequencing studies demonstrate that such deletions are more prevalent than any mutations of specific coding genes and many of these deletions have been linked to either ERG positive (i.e. PTEN and 3p13) or ERG negative cancers (i.e. 6q15 and 5q23). That high HOOK3 expression is linked to a higher prevalence of all analyzed deletions highlights a possible involvement of HOOK3 on mechanisms regulating genomic integrity. This is consistent with earlier work demonstrating that HOOK3 is relevant for proper function of the centrosomes, as it is essential for transport and dynamic assembly of centrosomal proteins [3,4]. Both knock down and ectopic overexpression of HOOK3 in cell line models resulted in compromised centrosomal functions [3], a fragmented Golgi apparatus, disrupted poorly organized microtubule network, and an increased fraction of cells with two or more nuclei [5]. In addition, centrosome abnormalities have been linked to aneuploidy in prostate cancer before [25].

Our in silico approach further demonstrated that HOOK3 overexpression is strongly linked to ERG activation. More than half of all prostate cancers, particularly those of young patients, carry gene fusions linking the androgen-regulated TMPRSS2 gene with the transcription factor ERG [14,26]. These genomic rearrangements result in an androgen-driven overexpression of ERG in affected cells [27] and, thus, altered expression of more than 1,600 genes in prostate epithelial cells [28]. It is unlikely, however, that high HOOK3 levels in ERG positive cancers are driven by direct activation, because the HOOK3 promoter region lacks ERG binding sites. Alternative explanations for the positive association of HOOK3 and ERG expression would include a direct or indirect interaction of these two proteins. It may, for example, be possible that HOOK3 –responsible for transport of centrosomal proteins—interacts with centrosomal proteins such as integrin-linked kinase (ILK), which is a relevant functional partner of ERG [29,30]. ILK and ERG have been shown to cooperatively drive malignant transformation and epithelial-mesenchymal transition in prostate cancer [31].

In summary, our study provides evidence that HOOK3 is an independent prognosticator in prostate cancer. We thus propose, that HOOK3 expression analysis has the potential for clinical routine application—either alone, or more likely, in combination with other biomarkers. Our large-scale tissue microarray approach will continue to prove highly instrumental for continuously identifying suitable prognostic biomarkers. Large scale molecular databases associated to large TMAs also enable limited functional analyses “in silico”.

## Supporting Information

S1 Table Clinico-pathological association of HOOK3 immunostaining in the ERG negative subset. (DOC)

S2 Table Clinico-pathological association of HOOK3 immunostaining in the ERG positive subset. (DOC)
