# Supplementary material for: High-Level HOOK3 Expression Is an Independent Predictor of Poor Prognosis Associated with Genomic Instability in Prostate Cancer
Source: PLoS One. 2015 Jul 31;10(7):e0134614. doi: 10.1371/journal.pone.0134614 (PMC4521853; doi:10.1371/journal.pone.0134614)
Supplement: S1 Table — (DOC) [file pone.0134614.s001.doc]

**S1 Table. Clinico-pathological association of HOOK3 immunostaining in the ERG negative subset.**

| **Parameter** | **Evaluable  (n)** | **HOOK3 (%)** | | | **p value** |
| --- | --- | --- | --- | --- | --- |
|  |  | **Negative** | **Low** | **High** |  |
| **All cancers** | 5,252 | 62 | 27 | 11 |  |
|  |  |  |  |  |  |
| **Tumor stage** |  |  |  |  |  |
| pT2 | 3,533 | 66 | 25 | 8.5 | <0.0001 |
| pT3a | 1,044 | 58 | 29 | 13 |  |
| pT3b-pT4 | 658 | 43 | 36 | 21 |  |
|  |  |  |  |  |  |
| **Gleason grade** |  |  |  |  |  |
| ≤3+3 | 1,108 | 80 | 16 | 4.2 | <0.0001 |
| 3+4 | 2,954 | 63 | 28 | 9.1 |  |
| 4+3 | 875 | 43 | 37 | 20 |  |
| ≥4+4 | 293 | 38 | 34 | 29 |  |
|  |  |  |  |  |  |
| **Lymph node metastasis** |  |  |  |  |  |
| N0 | 3,028 | 57 | 30 | 13 | <0.0001 |
| N+ | 300 | 39 | 35 | 26 |  |
|  |  |  |  |  |  |
| **Preoperative PSA level (ng/ml)** |  |  |  |  |  |
| <4 | 556 | 60 | 28 | 12 | 0.83 |
| 4-10 | 3,099 | 62 | 27 | 11 |  |
| >10-20 | 1,138 | 62 | 27 | 11 |  |
| >20 | 412 | 59 | 28 | 13 |  |
|  |  |  |  |  |  |
| **Surgical margin** |  |  |  |  |  |
| negative | 4,180 | 63 | 27 | 10 | 0.01 |
| positive | 983 | 59 | 28 | 13 |  |
