# Supplementary material for: High-Level HOOK3 Expression Is an Independent Predictor of Poor Prognosis Associated with Genomic Instability in Prostate Cancer
Source: PLoS One. 2015 Jul 31;10(7):e0134614. doi: 10.1371/journal.pone.0134614 (PMC4521853; doi:10.1371/journal.pone.0134614)
Supplement: S2 Table — (DOC) [file pone.0134614.s002.doc]

**S2 Table. Clinico-pathological association of HOOK3 immunostaining in the ERG positive subset.**

| **Parameter** | **Evaluable (n)** | **HOOK3 (%)** | | | **p value** |
| --- | --- | --- | --- | --- | --- |
|  |  | **Negative** | **Low** | **High** |  |
| **All cancers** | 4,118 | 26 | 49 | 26 |  |
|  |  |  |  |  |  |
| **Tumor stage** |  |  |  |  |  |
| pT2 | 2,452 | 29 | 49 | 22 | <0.0001 |
| pT3a | 1,097 | 20 | 51 | 29 |  |
| pT3b-pT4 | 552 | 20 | 42 | 38 |  |
|  |  |  |  |  |  |
| **Gleason grade** |  |  |  |  |  |
| ≤3+3 | 858 | 41 | 48 | 12 | <0.0001 |
| 3+4 | 2,450 | 24 | 50 | 27 |  |
| 4+3 | 626 | 14 | 48 | 38 |  |
| ≥4+4 | 161 | 19 | 40 | 42 |  |
|  |  |  |  |  |  |
| **Lymph node metastasis** |  |  |  |  |  |
| N0 | 2,358 | 22 | 49 | 29 | 0.02 |
| N+ | 266 | 19 | 44 | 37 |  |
|  |  |  |  |  |  |
| **Preoperative PSA level (ng/ml)** |  |  |  |  |  |
| <4 | 555 | 27 | 49 | 23 | 0.45 |
| 4-10 | 2,516 | 25 | 49 | 26 |  |
| >10-20 | 746 | 24 | 48 | 28 |  |
| >20 | 251 | 28 | 46 | 26 |  |
|  |  |  |  |  |  |
| **Surgical margin** |  |  |  |  |  |
| negative | 3,240 | 26 | 50 | 25 | 0.015 |
| positive | 800 | 26 | 45 | 29 |  |
